# Automated prediction of lattice parameters from X-ray powder diffraction patterns

**DOI:** 10.1107/S1600576721010840

**Published:** 2021-11-30

**Authors:** Sathya R. Chitturi, Daniel Ratner, Richard C. Walroth, Vivek Thampy, Evan J. Reed, Mike Dunne, Christopher J. Tassone, Kevin H. Stone

**Affiliations:** a Materials Science and Engineering, Stanford University, Stanford, CA94305, USA; b SLAC National Accelerator Laboratory, Menlo Park, CA 94025, USA

**Keywords:** analysis automation, machine learning, powder diffraction, indexing

## Abstract

A key step in the analysis of powder X-ray diffraction (PXRD) data is the accurate determination of unit-cell lattice parameters. This step often requires significant human intervention and is a bottleneck that hinders efforts towards automated analysis. This work develops a series of one-dimensional convolutional neural networks (1D-CNNs) trained to provide lattice parameter estimates for each crystal system. A mean absolute percentage error of approximately 10% is achieved for each crystal system, which corresponds to a 100- to 1000-fold reduction in lattice parameter search space volume. The models learn from nearly one million crystal structures contained within the Inorganic Crystal Structure Database and the Cambridge Structural Database and, due to the nature of these two complimentary databases, the models generalize well across chemistries. A key component of this work is a systematic analysis of the effect of different realistic experimental non-idealities on model performance. It is found that the addition of impurity phases, baseline noise and peak broadening present the greatest challenges to learning, while zero-offset error and random intensity modulations have little effect. However, appropriate data modification schemes can be used to bolster model performance and yield reasonable predictions, even for data which simulate realistic experimental non-idealities. In order to obtain accurate results, a new approach is introduced which uses the initial machine learning estimates with existing iterative whole-pattern refinement schemes to tackle automated unit-cell solution.

## Abstract 2

A method is introduced to determine lattice parameters using machine learning. Analysis is presented of the impact of experimental conditions on machine learning prediction, and possibilities for automated unit-cell solution are explored.

## 1. Introduction

Powder diffraction is a powerful technique for studying materials and has applications across a wide range of scientific areas. With the development of dedicated powder diffraction instruments at high-flux synchrotron beamlines, along with the development and widespread adoption of fast large-area detectors, data rates for powder diffraction have exploded. Development of high-throughput experimental setups and a greater emphasis on *in situ* and *operando* methods have compounded this problem. Characterizing materials as they are forming, or their structural response under intended operation, is crucial to design materials across application spaces that address pressing problems from energy security to domestic manufacturing (Krishnadasan *et al.*, 2007 ▸; Ren *et al.*, 2018 ▸). However, these types of measurements collect massive data sets and have already outpaced the capabilities of experimentalists to analyze these data manually (Blaiszik *et al.*, 2019 ▸). The time lag between collecting and analyzing data precludes the possibility of actionable information that can guide experimental design in real time. Fast automated data analyses are required which work on the timescale of an experiment, often seconds or minutes, to guide the next measurement towards the most informative one. The ability to parse massive data sets, recognize patterns not discernible by humans, accelerate data interpretation and provide real-time feedback to enable smart data collection is going to be an indispensable component of future materials research.

In this work, we focus on the problem of automatic analysis of powder X-ray diffraction (PXRD) data using a combination of machine learning (ML) and classical pattern-fitting approaches. A PXRD pattern is the result of three-dimensional atomic structure information condensed to a single dimension. The observed peaks are determined by the unit-cell structure (lattice parameters), symmetry constraints (space group) and atomic positions within the unit cell. The goal of a PXRD experiment, then, is the determination of these parameters. This experiment is particularly well suited to ML approaches, as the data can be readily simulated from known parameters, large databases of previously solved structures are available and patterns can be simulated on the basis of purely hypothetical materials.

Much previous work has focused on the problems of space-group and crystal-system prediction. Park *et al.* (2017 ▸) trained convolutional neural networks (CNNs) on simulated powder patterns, based on structural information contained in the Inorganic Crystal Structure Database (ICSD; https://icsd.fiz-karlsruhe.de/index.xhtml), to predict the correct crystal system and space group for a given material. The authors achieved an accuracy of 84% for the space-group task and an accuracy of 95% for the crystal-system task on simulated testing data. Subsequent analyses focused on the generalization gap between training on simulated data and testing on experimental data. Vecsei *et al.* (2019 ▸) developed fully connected architectures for the same problem which yielded superior generalization on their experimental data. Oviedo *et al.* (2019 ▸) developed a number of different models, including random forests, support vector machines, multilayer perceptrons and CNNs to predict space groups and dimensionality for thin-film perovskite structures. For their data, CNN models, trained on a combination of simulated and modified experimental data, were most effective. In particular, the approach used a physics-based augmentation scheme in order to correct for strain and preferred orientation in thin films (Oviedo *et al.*, 2019 ▸). More recent work has focused on developing extremely randomized trees for more interpretable ML predictions (Suzuki *et al.*, 2020 ▸) and on methods to emphasize differences between patterns with closely related space groups (Tiong *et al.*, 2020 ▸). Similar types of classification analysis have also occurred in the fields of electron diffraction (Aguiar *et al.*, 2019 ▸), single-crystal X-ray diffraction (Souza *et al.*, 2019 ▸) and neutron diffraction (Garcia-Cardona *et al.*, 2019 ▸). In addition, ML methods have been applied to tasks such as phase mapping (Utimula *et al.*, 2020 ▸; Stanev *et al.*, 2018 ▸; Long *et al.*, 2009 ▸), phase quantification (Lee *et al.*, 2020 ▸; Szymanski *et al.*, 2021 ▸), rapid database identification (Wang *et al.*, 2020 ▸), and automatic peak alignment for sequential data (Guccione *et al.*, 2018 ▸).

In this work, we present an ML approach for predicting lattice parameters from raw PXRD patterns. ML has previously been applied to tackle the unit-cell indexing problem (Habershon *et al.*, 2004 ▸). Similarly to traditional indexing approaches, the method applied by Habershon and co-workers requires the explicit extraction of the first 20 peak positions prior to making a prediction. Furthermore, since the lowest 20 reflections are needed, deviations away from this set arising from artifacts such as missing peaks and impurities can greatly damage the ML prediction. The key distinction in our approach is that our procedure is fully automated and can make predictions directly on raw intensity arrays, without the need for peak finding. We bypass the peak extraction step and automatically couple our ML predictions to a globally optimized whole-pattern fitting approach. We hope that this strategy might help enable a fully automated approach to powder diffraction pattern indexing suitable for the ever-increasing rate of data generation.

Other related work focuses on the analysis of particular materials systems with only small changes in composition and atomic positions (Garcia-Cardona *et al.*, 2019 ▸; Doucet *et al.*, 2020 ▸; Dong *et al.*, 2021 ▸). More recently, deep ensemble CNNs have been used to predict phase, symmetry and lattice parameters for an Ni–Pd/CeO_2_–ZrO_2_/Al_2_O_3_ multiphase system and have achieved results comparable to Rietveld refinement (Dong *et al.*, 2021 ▸). In contrast to these two approaches, our work seeks to be agnostic to particular material systems and instead to be able to yield lattice parameter estimates for any given crystalline material. Specifically, we are interested in understanding how well an ML approach for lattice parameter prediction can work without incorporating prior knowledge. In order to understand the motivation for using ML for this task, it is worth reviewing the classical methods of analysis.

Traditionally, lattice parameter calculation employs three steps: peak finding, peak indexing and refinement. The peak-finding step identifies the position of observed peaks in the diffraction pattern. The indexing step assigns Miller indices to each peak and obtains potential unit-cell assignments. Finally, the refinement step improves the estimate by minimizing a mean-squared-error loss between the experimental and calculated data. For clean high-resolution single-phase data with 20–25 non-overlapping reflections, it is sometimes possible to automate these three steps. A general peak-finding algorithm can select *d* spacings, and standard auto-indexing and refinement methods automatically solve the unit-cell structure (Visser, 1969 ▸; Coelho, 2018 ▸; Boultif & Louër, 1991 ▸; Altomare *et al.*, 2009 ▸; Le Bail, 2004 ▸).

However, data from real experiments can often be noisy and contain highly overlapped regions from data with more than one phase. In these situations, it is difficult to determine the number of reflections in a given region, which makes accurate determination of peak locations challenging. Furthermore, for multiphase samples, assigning peaks to their correct corresponding phase is a significant challenge. In fact, for many low-symmetry crystals, automatic peak finding can even fail on simulated data (Park *et al.*, 2017 ▸). In these cases, peak finding and indexing often become a human-supervised procedure, which impedes progress towards continuous-analysis paradigms.

In this work, we train CNNs to predict lattice parameters for each crystal system on the basis of data from the Cambridge Structural Database (CSD; Groom *et al.*, 2016 ▸) and ICSD (Hellenbrandt, 2004 ▸). Together, these data sets contain crystal structures with a large range of lattice parameters and whose structures involve different types of bonding and compositions. The ML approach to the indexing problem is fundamentally different from the classical methods. Instead of assigning (*hkl*) indices to each peak, ML methods rely on learned correlations to make predictions. These relationships are found by looking at a vast database of different crystal structures. ML approaches have the potential to outperform conventional methods in at least two key areas:

(i) Stability to noise. ML methods learn patterns that are characteristic of particular lattice parameters. This opens the possibility of leveraging prior knowledge of crystal systems to condition predictions on noisy, overlapped and multiple-phase data.

(ii) Speed. The prediction process can be run in real time during experimental data acquisition, as inference with pre-trained models is faster than the time required to acquire data.

In this paper, we present an ML method which can be used to predict the lattice parameters from generic raw PXRD patterns. In doing so, we also highlight various challenges with such an approach and, where possible, suggest areas for future improvement. As part of our analysis, we analyze various experimental conditions which are known to present challenges to the indexing process, including baseline noise, zero-offset error, peak broadening, multiple impurity phases and intensity modulations due to preferred orientations. We use data modification strategies to mitigate these problems and quantify the improved performance of the ML models. We validate our results on simulated data from the ICSD and CSD and on a small selection of synchrotron data from Beamline 2-1 at the Stanford Synchrotron Radiation Light Source (SSRL). Finally, we present a proof-of-concept approach by combining our initial lattice parameter estimates with the *Lp-Search* algorithm, a recently introduced whole-pattern fitting approach which can refine unit-cell structures without extracting *d* spacings (Coelho, 2017 ▸). Our intention is that this work, and the associated code base, will be valuable to the community in providing a guide for future ML-based indexing for generic PXRD patterns.

## 2. Analysis of simulated data from the ICSD and CSD

### 2.1. ICSD and CSD combined data

**Figure 1:**
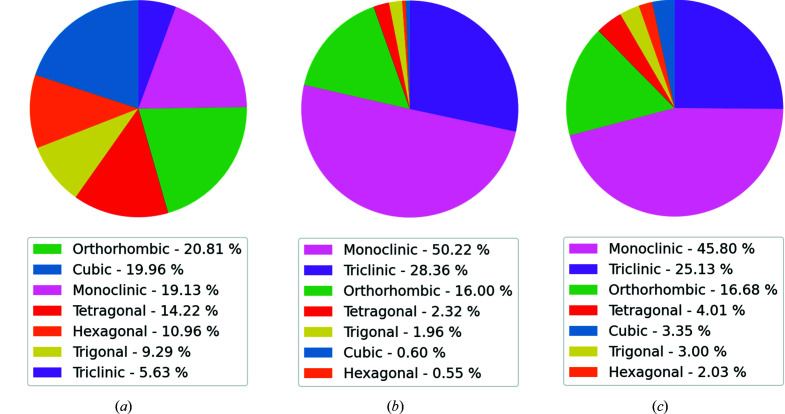
Visualizations of data distribution by crystal system in (*a*) the ICSD, (*b*) the CSD, and (*c*) both the ICSD and the CSD. These two databases exhibit complementary distributions which justifies the choice to combine them.

We begin by considering the problem of predicting lattice parameters under conditions of perfect information. Lattice parameters are specified by the Bragg equation and can be determined from the peak positions in a PXRD pattern alone. Intensities are modulated by other factors, such as the crystal structure factor or sample and detector effects. For this reason, it should be possible to combine information from different types of databases to improve the prediction of lattice parameters. Here we simulate PXRD data from the ICSD, a database for inorganic crystals which is significantly populated with high-symmetry structures with relatively small lattice parameters. We also simulate data from the CSD, a repository for small-molecule organic and metal–organic crystal structures. This database is significantly populated with lower-symmetry structures and contains some entries with very large lattice parameters. The full details of the PXRD simulations are described in the supporting information (Section S2.1). Together, these two databases are highly complementary and combining them increases the diversity of the data (Fig. 1 ▸). The combined data set has 961 960 entries with at least 15 000 patterns for each crystal system (Table 2).

Our approach is to train one-dimensional convolutional neural networks (1D-CNNs) on raw intensity arrays and without any direct feature engineering. We approach this task as a supervised regression problem where the labels are the numerical values of the lattice parameters which, for each structure, are contained in either the ICSD or the CSD. For each set of labels, there is a corresponding PXRD pattern which is denoted as the input. At a high level, a supervised ML regression model can be interpreted as a nonlinear map from the input data (PXRD pattern) to the predicted output (lattice parameters). We train seven separate models corresponding to each one of the seven crystal systems. At test time, the correct crystal system is specified and the corresponding 1D-CNN is used to make predictions of the values of the lattice parameters for new PXRD patterns. In this work, we assume that the crystal system is provided beforehand. In doing so, we assume that in a real *operando* implementation this information could be obtained by leveraging the highly accurate 1D-CNN models recently developed for crystal-system classification (Park *et al.*, 2017 ▸; Vecsei *et al.*, 2019 ▸; Oviedo *et al.*, 2019 ▸; Suzuki *et al.*, 2020 ▸; Tiong *et al.*, 2020 ▸). Here, we see our work as highly complementary to other ML-based approaches in the community.

The choice to train models conditioned on each crystal system has two primary motivations. Firstly, each crystal system has a different number of independent lattice parameters which generate the data. Secondly, indexing is not unique and therefore it is possible for a crystal to be indexed in more than one crystal system; these types of non-one-to-one tasks are typically more challenging from an ML perspective. Note that CNN models trained for each extinction class or space group should perform even better than models for each crystal system (Habershon *et al.*, 2004 ▸). Although we explore this idea further in the supporting information (Section S1.3.1), the primary focus of the paper is on crystal systems, since we wanted to keep our analysis as general as possible.

For this reason, we also opt to keep the same 1D-CNN architecture for every analysis presented in the paper (Table 1 ▸). Here, the connectivity between all the layers is the same, but the weights will change according to the data. Briefly, the layers denoted Conv1D correspond to convolutional layers that learn a series of filters which are able to process PXRD patterns. Each convolutional layer has an activation function which is a nonlinear transformation used to increase the representational power of the neural network. Early convolutional layers generally learn simple features such as identifying regions of large intensity variation including peaks and valleys. The MaxPooling1D operation downsamples the input by performing the maximum operation. The reason for these layers is to consolidate information from various learned filters and represent them in a lower-dimensional space. Finally, the Dense layers correspond to simple fully connected layers which are generally useful for nonlinear regression tasks. The details of this model are described in further detail in Section S2.2. An extensive review of CNNs is provided by Rawat & Wang (2017 ▸).

We train baseline 1D-CNN models for each crystal system and report the testing mean absolute percentage error (MAPE) for the prediction of the *a*, *b* and *c* lattice parameters (Table 2 ▸). We consider the cases where the PXRD pattern is simulated from 0 to 30° and 0 to 90° in 2θ with a wavelength of 1.54056 Å. As a baseline, we compare the predictions against a null model which uses the mean lattice parameters in the full data set as the prediction output. A null model is important to this analysis because it shows whether an ML approach is able to learn any information about the mapping between PXRD patterns and lattice parameters. Importantly, the interpretation is not that any improvement over the null model is intrinsically meaningful, but rather that the degree of relative improvement over the null model can give a sense of how well the ML approach works. For instance, we seek to avoid claiming that the ML model performs well in crystal systems where it is simply the case that the data have a narrow distribution of lattice parameter values. The motivation for choosing the mean null model is that it does not require any peak-finding operations in order to provide an estimate. We also consider other null models in Section S1.3 and find that the various null models perform similarly, with no individual model the clear winner for all crystal systems.

From this analysis, we are able to predict lattice parameters for all crystal systems with roughly 10% MAPE for both angular ranges (Table 2 ▸). We quantify the extent to which the ML models outperform the null model by taking the ratios (ratio 1 and ratio 2) of the relevant MAPEs. Clearly, the ML predictions far outperform the null models, and this highlights the predictive potential of ML for the parameter estimation task. In addition, the models perform similarly for both angular ranges, which suggests that it might not be necessary to include higher-angle data. This finding agrees with the conventional wisdom that a smaller range, containing 20–30 peaks, is generally sufficient to index a powder pattern. Nevertheless, we focus on the 0–90° range in this work in order to avoid needing to specify a range containing a certain number of peaks.

Note that we were not able to predict accurately the three angle parameters, α, β and γ. For these cases, our predictions were comparable to a null prediction based on the mean angle parameters in the data set. There are a few possible reasons for this result which are considered further in Section S1.2. However, this result only affects the predictions for the monoclinic and triclinic crystal systems, since angle information is implicitly considered by training independent models for each crystal system. We recognize that the lack of angle information does hinder the predictive ability for low-symmetry structures. However, in Section 3.2 we show that, in some cases, a coupled scheme involving ML and automated refinement can be used to recover the angles for the monoclinic and triclinic systems.

#### 2.1.1. Training and testing on modified data

Real-life experimental conditions introduce various deviations to PXRD patterns simulated from crystal structure factors. To develop effective ML algorithms for automatic prediction, it is critical to determine possible experimental non-idealities in the input data that significantly affect predictive performance. On the basis of experimental guidelines and previous work (Oviedo *et al.*, 2019 ▸; Park *et al.*, 2017 ▸), we consider the effect of the following modifications due to experimental non-idealities: peak broadening, baseline noise, random intensity modulation, detector zero shifting and the presence of multiple unknown phases. The motivation for and details of these experimental modifications are described in Section S2.3.

**Figure 2:**
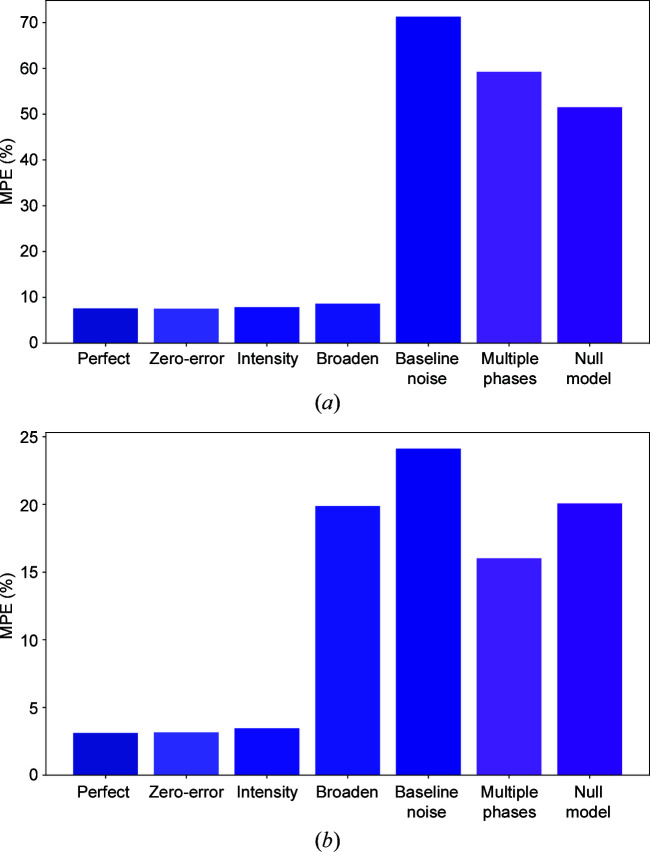
The effect of including experimental modifications in testing data on models trained without including any experimental modifications. Results are shown for unit-cell length predictions for (*a*) the cubic and (*b*) the triclinic crystal systems. The modifications studied correspond to zero error, intensity modulation, Gaussian broadening, Gaussian baseline noise and multiple impurity phases. Perfect refers to unmodified data and null refers to prediction using the mean lattice parameters of the data set. Zero error and intensity modulation have little effect on ML prediction. On the other hand, baseline noise and multiple phases are particularly damaging modifications.

First, we consider the situation where our models are trained on clean (no experimental modifications) simulated data and tested on simulated data containing one of the aforementioned experimental modifications. Fig. 2 ▸ shows the effect of different experimental modifications for a representative high-symmetry class (cubic crystals) and a representative low-symmetry class (triclinic crystals); the full data for all crystal systems are also presented in Table 3 ▸.

In both cases, random intensity modulation and zero shifting clearly have little effect on the model prediction. Here, the performance, as quantified by MAPE, is similar to the prediction on perfectly clean data. The results for the intensity modulation experiment indicate that the models are correctly learning the physics that lattice parameter prediction should be dependent on peak location and not intensity.

We note that the method for modifying the intensities does not entirely capture the process of preferred orientation effects in PXRD patterns. It is possible that a more physically realistic model for intensity modulation, which is dependent on Miller indices, might be detrimental to the ML performance. However, we can be confident that, at least for small modulations in intensity, the ML performance should be relatively stable. Although outside the scope of the present work, to study the effect of strong orientation effects it will be important to incorporate a more realistic physical model.

The results for the zero-error experimental modification indicate that the 1D-CNN models are largely invariant to small total translations of the input image. In other words, for small offsets, only relative distances between peaks matter. This is an unsurprising result, as translational invariance is one key feature of CNN approaches. On the other hand, it is evident that baseline noise and the presence of multiple phases are extremely damaging to model prediction (Table 3 ▸). Under these conditions, the prediction is comparable to a null model which predicts lattice parameters based on the mean lattice parameters of the full data set. This indicates that the initial unmodified models are highly sensitive to the presence of small additional peaks and are not suitable for application to real data sets. Another interesting result is that modification with peak broadening affects the triclinic system far more than the cubic crystal system (Fig. 2 ▸). This is probably because the triclinic system has a large number of peaks (due to lower symmetry) which become highly overlapped with increased broadening. This trend of worsening performance for lower-symmetry crystals with peak broadening also holds for other crystal systems (Table 3 ▸).

To improve the resilience of the model to experimental modifications, we analyzed the effect of including modified examples in the training data. For example, to improve the performance of our model against baseline noise on the triclinic system, we trained a 1D-CNN with data that had variable baseline noise. We focus only on improving the performance against multiple phases, baseline noise and peak broadening, since random intensity modulation and zero shifting have little impact on predictive performance (Table 3 ▸).

**Figure 3:**
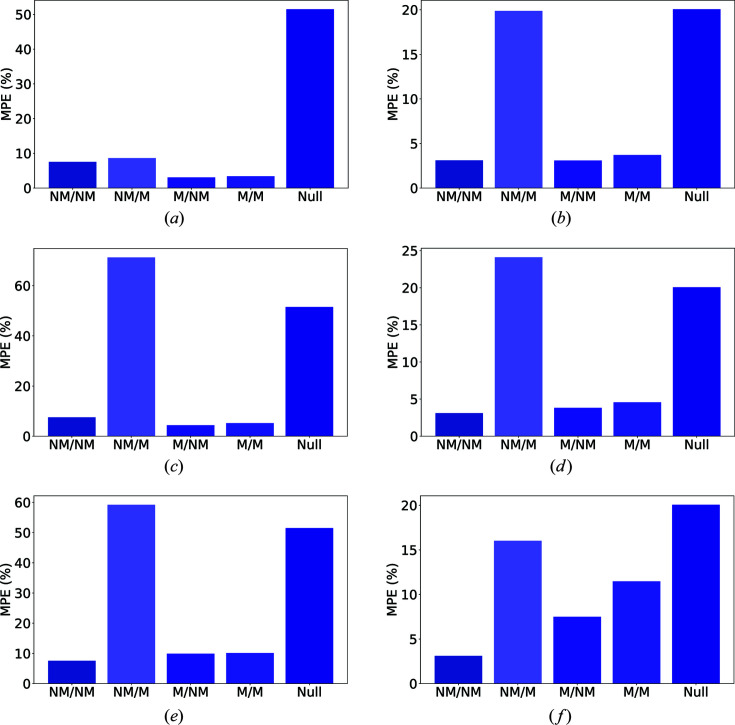
The impact of (*a*), (*b*) broadening, (*c*), (*d*) baseline noise and (*e*), (*f*) multiple impurity phases on ML predictions for unit-cell lengths for (left-hand column) cubic and (right-hand column) triclinic crystal systems. NM refers to a pattern with no modifications and M refers to a pattern with the corresponding experimental modification. The notation *A*/*B* indicates training with modification *A* (NM or M) and testing with modification *B* (NM or M). The null column is a prediction based on the mean lattice parameters of the data set. The performance of ML models is greatly improved when training and testing with modifications (M/M) relative to training on unmodified data and testing on modified data (NM/M).

For each type of modification, we consider four tests which constitute all possible choices of training and testing on unmodified (NM) and modified (M) data. The results are shown graphically in Fig. 3 ▸ for the cubic and triclinic crystal systems. More complete information for all crystal systems is presented in Tables 4 ▸ and 5 ▸.

For the experimental broadening condition, we find that it is possible to stabilize greatly the predictions for all crystal systems. For example, the MAPE for the triclinic system is more than four times lower for training and testing on modified data versus training on unmodified data and testing on modified data [Fig. 3 ▸(*a*)]. Interestingly, we find that training on data with broadening even helps prediction on unmodified data; this is indicative of classical augmentation improvement effects that are often seen in training ML models (Perez & Wang, 2017 ▸). In short, our analysis suggests that it should be relatively easy to stabilize predictions against peak broadening by incorporating the broadening modification into the training set.

Incorporating baseline noise into the training set also greatly reduces the testing MAPE [Fig. 3 ▸(*b*) and Table 4 ▸]. However, for a number of crystal systems, the performance is not as good as the default of training and testing without baseline noise. Furthermore, it appears that a model trained on modified data and applied to data without any modification yields a slightly worse prediction than a trained unmodified model (Table 5 ▸). We believe that the reason for these observations is that a model trained on baseline noise becomes less sensitive to low-intensity peaks. This would primarily affect high-angle/high-*q* data. Therefore, training using baseline noise should help the performance on a modified test set, but, since there is a loss of information in training, the model performs slightly worse on clean data.

We also analyzed how the ML performance changes as the baseline noise level is increased, reducing the number of visible peaks. Here, a peak is defined as no longer visible if it has lower intensity than the surrounding baseline noise. As a representative example, we show the results for the tetragonal crystal system in Table 6 ▸. Although the performance decreases, even at a noise level of 0.1 (10% noise relative to the largest peak) where only 33% of peaks are visible, the predictions are significantly better than those of the null model.

Finally, we consider the impact of including multiple impurity phases on model testing performance [Fig. 3 ▸(*c*) and Tables 4 ▸ and 5 ▸]; here we consider the case where we have up to three low-phase-fraction impurities (Section S2.3). We note similar trends to the baseline noise case, but the magnitude of the deteriorating effect is more pronounced. This is consistent with the intuition that the presence of multiple impurity phases is one of the most challenging obstacles in classical indexing procedures. Interestingly, we still get reasonable predictions, even without filtering the impurities, especially for higher-symmetry structures. Furthermore, we emphasize that our analysis incorporates extremely stringent tests for impurity peaks at a level far beyond that of conventional indexing. On average, a given pattern might be corrupted by a large number of peaks with non-negligible intensities (Table 7 ▸). In general, the problem of solving unit cells in the presence of multiple phases is an important unsolved problem in powder diffraction. Our results indicate that ML approaches can provide pathways to estimating the lattice parameters of PXRD patterns in the presence of multiple small impurity phases.

Overall, the results of this analysis of possible experimental modifications show that it is essential to incorporate appropriate modified data into ML training sets. Furthermore, proper modification strategies can substantially recover lost predictive power.

### 2.2. Percentage within bound metric

In this section, we introduce the percentage within bound (PWB) metric to analyze further the performance of the ML predictions. The PWB is the percentage of test examples which have all three lattice parameters within a given MAPE (Section S2.2). Concretely, a PWB10 metric measures the likelihood that all three lattice parameter predictions are within 10% of their true values. We believe this is a better (although harsher) metric than MAPE and is more suitable for assessing performance. For this analysis, we train models on ICSD/CSD data with all data augmentations mentioned in Section 2.1.1. The performance on a test set of ICSD/CSD data for each crystal system compared with a null prediction based on the mean lattice parameters of the data set is shown in Table 8 ▸. For testing on ICSD/CSD data, for every bound, the ML prediction significantly outperforms the null prediction.

## 3. Perspectives on automated analysis

So far, we have shown that it is possible to estimate lattice parameters on simulated data with approximately 10% MAPE (Table 2 ▸). Although this is a promising result for an ML approach, these results do not solve the unit cell from a practical viewpoint. Generally, in order to solve a unit cell, it is necessary to estimate the lattice length parameters *a*, *b* and *c* to within 0.1–0.01 Å and the angles to within 0.1°. In this section we first quantify the extent to which ML predictions reduce the search space needed to find the true lattice parameters. Following this, we present a coupled scheme which uses ML estimates and iterative whole-pattern fitting to solve unit cells automatically. Finally, we apply this methodology to a small data set from Beamline 2-1 at the SSRL.

### 3.1. Volumes of parameter search space

Using an ML prediction, we are able to reduce greatly the volume of parameter search space around the true values. Our baseline range for the three lattice parameters is between 2 and 2*d*_max_ Å, where 2*d*_max_ represents an upper bound on the largest lattice parameter, and this estimate forms a cube in lattice parameter space. *d*_max_ is calculated by using the knowledge of the correct crystal system to solve directly for the largest *d* spacing (Section S2.5). This bound was chosen as it is used as the default bound for the whole-pattern fitting approach described in Section 3.2. The lower bound was chosen to include all structures from the testing sets. Real indexing strategies often contain other constraints, such as *a* ≤ *b* ≤ *c*, on the search space volume. These strategies reduce both the ML and the default bound and are hence not considered here. We calculate the percentage of the testing data set which falls within 10 and 5% bounds (PWB5 and PWB10) around the ML estimates, as well as the corresponding reductions in search space volume for these bounds, VR_5_ and VR_10_ (Table 9 ▸). The volume metrics are calculated as ratios of the baseline search space volume to the ML search space volume, averaged over all the testing set predictions (Section S2.5). Note that the predicted ML volume is a rectangular prism, as opposed to the 2–2*d*_max_ cubic volume for the baseline.

Unsurprisingly, the reduction in search space is much more pronounced for lower-symmetry systems than for higher-symmetry systems. For example, VR_10_ exceeds a factor of 1000 for low-symmetry crystals (Table 9 ▸). In this work, the ML search space volume only takes into account predictions of the lattice length parameters. We expect that, with the development of better models which can accurately predict lattice angle parameters, the relative difference between the baseline search space and the ML search space volumes will be even larger for the triclinic and monoclinic crystal systems. These results will probably be generally useful to the indexing community at large, since popular indexing approaches such as trial and error and Monte Carlo search (Altomare *et al.*, 2009 ▸; Le Bail, 2004 ▸), the dichotomy method (Boultif & Louër, 1991 ▸), and singular value decomposition (Coelho, 2003 ▸) could directly incorporate these restricted ranges into their analyses. This would be a trivial addition to these algorithms as each optionally allows for constrained search within specified unit-cell ranges.

### 3.2. Whole-pattern fitting using ML initial guess

In this section, we combine our ML estimates with *Lp-Search*, a recently developed whole-pattern refinement method based on Pawley refinement (Coelho, 2017 ▸). The guiding motivation for using *Lp-Search* is that it has wider minima for the objective loss function than in Pawley refinement and can work with less accurate initial parameter estimates. *Lp-Search* often performs quite well on simulated data and can sometimes solve unit cells using the default 3–2*d*_max_ parameter ranges. We present three case studies comparing ML+*Lp-Search* with default *Lp-Search* and analyze the results in terms of speed and convergence. The PXRD patterns we consider here correspond to a high-symmetry cubic structure, a low-symmetry triclinic structure and a hexagonal dominant zone problem (Table 10 ▸).

#### 3.2.1. Example 1: case study of a high-symmetry system

We first consider Example 1 (Baffier & Huber, 1969 ▸), which is the simple case of indexing a crystal with cubic symmetry (Table 10 ▸). Here the true lattice parameter *a* has a value of 8.292 Å and the ML prediction yields an estimate of 8.666 Å. For the ML+*Lp-Search* method, we initialize *Lp-Search* with ML lattice parameter ranges that are within 10, 20 and 50% of the predicted values. These estimates are fed into the *Lp-Search* algorithm, along with the correct crystal system, and the average times taken to converge to the true lattice parameters are recorded. In addition, we report the fraction of times a full *Lp-Search* minimization converges to the correct answer within 50 000 iterations. The minimization was also performed using the *Lp-Search* default range of (3–2*d*_max_) for each lattice parameter (Table 11 ▸).

In this example, while ML+*Lp-Search* yields a reduction in search space and is faster than default *Lp-Search*, the corresponding volume reduction and speedup are modest. Furthermore, in both cases, the minimizations converge to the true answer in every minimization. This is an unsurprising result as the cubic system is generally easy to index since the search space is one dimensional.

#### 3.2.2. Example 2: case study of a low-symmetry system

Next, we consider a low-symmetry triclinic structure (Odermatt *et al.*, 2005 ▸) with dissimilar values for the lattice parameters (Example 2; Table 10 ▸). The predicted and true *a*, *b* and *c* lattice parameters are 11.559548, 13.5853405 and 38.7705 Å and 11.2927, 13.455 and 37.9436 Å, respectively. The corresponding percentage converged and average times taken to converge are shown in Table 12 ▸. For this example, the angular lattice parameters were initialized to be 90° in 2θ with an allowable range of [60°, 120°] 2θ in the *Lp-Search* procedure.

In this case, ML+*Lp-Search* is much faster than default *Lp-Search* and converges every time. As expected, the tighter the ML bound, the faster the ML+*Lp-Search* method reaches convergence. This speedup can be directly attributed to the large reduction in search space using our initial ML estimates. Note that the ML was useful for this problem even in the absence of predictions for α, β and γ. We expect that future work on accurate prediction of lattice angle parameters will further significantly accelerate the method.

#### 3.2.3. Example 3: case study of a dominant zone system

Finally, in Example 3 (Huang *et al.*, 2018 ▸) we investigate an example of a structure which exhibits a dominant zone problem (Table 10 ▸). In this situation, one lattice parameter is much larger than the others and therefore the first set of peaks correspond to just the largest lattice parameter. These problems are typically challenging for all conventional indexing programs, as well as for *Lp-Search* (Coelho, 2017 ▸). The corresponding percentage converged and average times to converge are shown in Table 13 ▸.

In this case, the ML+*Lp-Search* method correctly determined the lattice parameters using a 10% bound in all of the minimizations; this situation corresponds to VR_10_ = 404. As the bound increases, the fraction of converged solutions decreases, while the average time taken to converge increases (Table 13 ▸). Notably, default *Lp-Search* did not converge to the correct lattice parameters in any of the 20 minimizations. Again, this result is attributable to the large reduction in search space for initial ML estimates. These results highlight that the ML+*Lp-Search* method has the potential to index structures, automatically, which are challenging for conventional methods.

In these three case studies, our analysis specifies *a priori* the bound that contains the true lattice parameters. This is not necessarily a problem as it is possible to try, iteratively, different bounds of increasing width. One avenue of future research will be to quantify the uncertainty of the ML prediction using probabilistic models to learn the appropriate bounds directly. Here, one approach could involve ensembling various 1D-CNN models and using the predicted 95% intervals as the *Lp-Search* bounds. In addition, although the time taken to make a prediction on lower-symmetry systems may appear relatively long, in practice this implementation could require far fewer than the 50 000 iterations used here. The case studies were run with 50 000 iterations in order to give the default *Lp-Search* range the maximum chance of converging. In addition, as *Lp-Search* is trivially parallelizable (Kirk & Wen-Mei, 2016 ▸), an implementation of this procedure at a beam­line could easily operate using a small cluster of CPU cores.

### 3.3. Quantifying necessary bounds for *Lp-Search*

**Figure 4:**
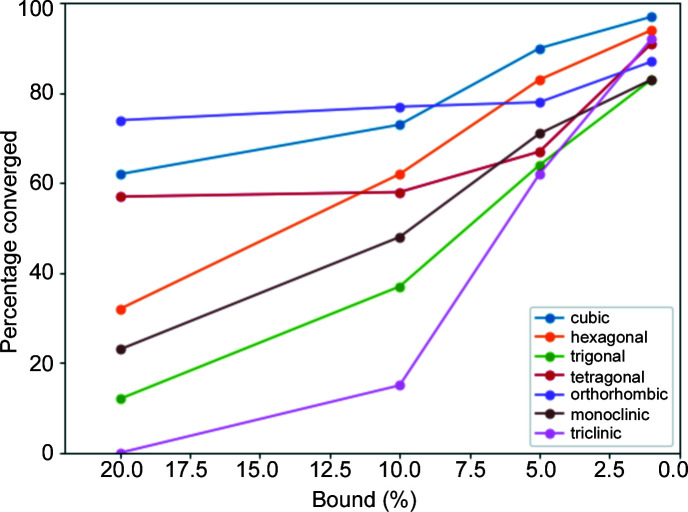
Percentage of times that *Lp-Search* converged to the correct answer as a function of percentage bound for 100 test samples for each crystal system.

In addition to the case studies on simulated data, we studied how tight the range for a prediction needs to be in order to converge with *Lp-Search* in just 1000 iterations for 100 samples from each crystal system (Fig. 4 ▸). Specifically, *a*_max_–*a*_min_, *b*_max_–*b*_min_, *c*_max_–*c*_min_, α_max_–α_min_, β_max_–β_min_ and γ_max_–γ_min_ were chosen as 1, 5, 10 and 20 percentage deviations from the true lattice parameters. In doing so, we quantify how good an ML design needs to be in order to converge reliably with *Lp-Search* on simulated data using a very small number of minimizations.

Our results indicate that ML predictions within 1–5% of the true lattice parameters are likely to converge automatically in a short number of *Lp-Search* iterations. The intuition for choosing a small number of *Lp-Search* iterations was to formulate the problem as an ML-guided local optimization problem which can be run on a single local CPU. However, it is certainly possible to use a greater number of minimizations. For a larger number of minimizations, we expect that the probability of convergence will increase monotonically for every bound threshold. Such an approach would enable more successful attempts for lower thresholds. Nevertheless, we hope that this analysis will be helpful in setting a concrete target for improvements on the approach presented here. For fast high-throughput experiments, we expect fully automated methods such as ML+*Lp-Search* to be quite valuable for indexing data with a single dominant phase.

### 3.4. Application to synchrotron data

Finally, we apply the models from Section 2.2 to experimental data collected from Beamline 2-1 at the SSRL. Since these data are collected at different wavelengths (typically 0.729 Å) from the training data, they are first linearly interpolated to fit the same *q* range as the training data. We present the ML unit-cell predictions (ML *a*, *b*, *c*), the ML+*Lp-Search* predictions (ML/*Lp* *a*, *b*, *c*) and ground-truth parameters from expert refinement in Table 14 ▸.

The ML+*Lp-Search* method performs reasonably well and, in the majority of high-symmetry cases, the procedure converges to the correct lattice parameters automatically. The performance in certain monoclinic low-symmetry cases, however, is not so good. We speculate that there are at least two reasons for this observation. First, the monoclinic system contains a large quantity of data from various extinction classes which might be confusing the ML predictions. This reasoning seems to be supported by our observations that it is possible to train better ML models when space-group information is utilized (Section S1.3.1). The second probable reason for worse performance in the monoclinic system is the lack of predictability of the angle using the ML approach. *Lp-Search* is given the full range for the possible angle and it is possible that this search space is too large for *Lp-Search* to converge in the specified number of iterations. Possible reasons for the difficulty in predicting the lattice angle parameters are detailed in Section S1.2. Also, for a few cases, the minimization converges to lattice parameters that are trivial multiples or divisors of the refined ground truth. These predictions are highlighted with an asterisk (*) in Table 14 ▸. In order to tackle this issue and to obtain the preferred higher-symmetry solutions, training models for each space group or extinction class will probably be necessary.

## 4. Conclusions

The ability to refine a unit cell without human supervision will help drive future work in the optimization of materials. In this work, we help build the framework necessary to realize this goal. By training deep convolutional neural networks on nearly a million unique PXRD patterns drawn from across the chemical spectrum, we are able to provide estimates of unit-cell lattice parameters for each crystal system. In doing so, we analyze key experimental non-idealities that might affect ML predictions and conclude that the presence of multiple phases, baseline noise and peak broadening are particularly damaging. Incorporating these experimental conditions into the training is absolutely necessary and, in many cases, can improve model prediction and stability.

We emphasize that our approach is independent of particular chemical environments and instead should apply to all crystalline systems at large. However, there are many situations in which the possible phases, elemental constituents and bonding are known. If the constituent elements are known, this can be used to construct a prior model for the neural network, since atomic features such as ionization energies and electronegativities are generally correlated with lattice length parameters (Li *et al.*, 2021 ▸). For instance, if the constituent elements and compositions can be obtained using another characterization method, a joint model can be trained over the elemental features and the PXRD patterns in order to yield better predictions. In addition, the modular nature of our work allows our models to be directly combined with other similar analyses for different types of characterization data in order to leverage multiple sources of information simultaneously (Aguiar *et al.*, 2020 ▸).

The primary focus of this work was predicting the lattice parameters of a dominant phase in the presence of relatively weak impurities. However, a more general and useful task is the prediction of lattice parameters for all sets of phases within a PXRD pattern. This task is ill-posed under the current formulation since only the label of the dominant phase is provided during training. It is also difficult to extend this directly to multiple phases because the labels would need to have variable dimensionality to account for the different numbers of phases. In order to train such an algorithm, a training set would need to be constructed which consists of linear combinations of phases and their corresponding lattice parameters. In this situation, the size of the data set scales combinatorially with the number of possible patterns. For the cases where all possible phases within the system are specified, either from theory or prior knowledge, it is possible to train such an algorithm and achieve successful results (Lee *et al.*, 2020 ▸; Dong *et al.*, 2021 ▸). Unfortunately, the more interesting case applies in the regime of materials discovery, where not all phases present are known beforehand. In order to approach this problem, additional information is needed. Specifically, if the system can be observed with different linear combinations of phases (*e.g.* through high-throughput sputtering or time-series experiments), it may be possible to utilize our algorithm on plausible reconstructed single phases obtained via non-negative factorization methods (Utimula *et al.*, 2020 ▸; Stanev *et al.*, 2018 ▸; Long *et al.*, 2009 ▸).

Finally, we have demonstrated that the initial parameter estimations can lead to a substantial reduction in search space volume around the true lattice parameters. We believe that these results, by themselves, are useful to the powder diffraction community due to their ease of integration with conventional indexing techniques such as singular value decomposition and the dichotomy method. Furthermore, we demonstrate that these estimates can be directly passed to whole-pattern refinement schemes in order to solve the unit cell for cases automatically. Future work on the angle prediction problem will probably increase the success rate for lower-symmetry materials. The significant reduction in search space volume enabled by lattice parameter prediction brings a corresponding acceleration of such whole-pattern refinement schemes. Full solutions are achievable on timescales suitable for feedback into the experimental process. Accelerated and fully automated analysis pipelines are a prerequisite for Bayesian optimization or reinforcement learning approaches, which will allow for the exploration of high-dimensional and complex materials parameter spaces becoming common for *operando* or *in situ* experimentation.

## 5. Code and data availablility and supporting information

Models for all modification experiments, the SSRL Beamline 2-1 data set and scripts to generate *Lp-Search* input files can be accessed at https://github.com/src47/DeepLPnet. Please contact the ICSD and CSD for access to the simulated structures described in this paper.

The supporting information contains details of additional analysis, methods and data. For further literature related to the supporting information, see Chollet (2015 ▸) and René de Cotret & Siwick (2017 ▸).

## Supplementary Material

Additional analysis, methods and data. DOI: 10.1107/S1600576721010840/vb5020sup1.pdf

## Figures and Tables

**Table 1:** The 1D-CNN architecture used for PXRD data sets The same architecture was used for all experiments, although the weights were allowed to vary according to the data.

Layer (type)	Output shape	No. of parameters
InputLayer	(9000, 1)	0
MaxPooling1D (pool size = 3)	(3000, 1)	0
Conv1D (kernel = 5, filter = 3, activation = ReLU)	(3000, 5)	20
Conv1D (kernel = 5, filter = 3, activation = ReLU)	(3000, 5)	80
MaxPooling1D (pool size = 2)	(1500, 5)	0
Conv1D (kernel = 10, filter = 3, activation = ReLU)	(1500, 10)	160
Conv1D (kernel = 10, filter = 3, activation = ReLU)	(1500, 10)	310
MaxPooling1D (pool size = 2)	(750, 10)	0
Conv1D (kernel = 15, filter = 5, activation = ReLU)	(750, 15)	765
Conv1D (kernel = 15, filter = 5, activation = ReLU)	(750, 15)	1140
MaxPooling1D (pool size = 3)	(250, 15)	0
Conv1D (kernel = 20, filter = 5, activation = ReLU)	(250, 20)	1520
Conv1D (kernel = 20, filter = 5, activation = ReLU)	(250, 20)	2020
MaxPooling1D (pool size = 2)	(125, 20)	0
Conv1D (kernel = 30, filter = 5, activation = ReLU)	(125, 30)	3030
Conv1D (kernel = 30, filter = 5, activation = ReLU)	(125, 30)	4530
MaxPooling1D (pool size = 5)	(25, 30)	0
Flatten	(750)	0
Dense (activation = ReLU)	(80)	60080
Dense (activation = ReLU)	(50)	4050
Dense (activation = ReLU)	(10)	510
Dense (activation = ReLU)	(3)	33

**Table 2:** MAPE for 1D-CNNs for each crystal system Null prediction refers to a MAPE prediction based on the mean lattice parameters of the data set. ML full corresponds to the prediction from the 1D-CNN models for 0–90° in 2θ. ML reduced corresponds to the prediction from the 1D-CNN models for 0–30° in 2θ.

Symmetry	Null prediction	ML prediction full	Ratio 1	ML prediction reduced	Ratio 2	Training data set size
Cubic	51.49	7.55	6.8	4.08	12.6	30 705
Hexagonal	47.37	7.35	6.4	8.94	5.3	17 842
Trigonal	46.58	15.72	3.0	15.30	3.0	25 784
Tetragonal	48.77	11.56	4.2	9.09	5.4	37 183
Orthorhombic	29.94	10.06	3.0	12.92	2.3	161 087
Monoclinic	24.76	11.79	2.1	11.23	2.2	445 708
Triclinic	20.06	3.11	6.5	2.68	7.5	243 651

**Table 3:** MAPE for 1D-CNNs trained on unmodified data and tested on data containing modifications Baseline noise, broadening and impurities damage ML performance, while intensity modulations and peak shifting have little effect. Null refers to predictions based on the mean lattice parameters of the data set. Perfect refers to training and testing on unmodified data and is intended as a control.

Symmetry	Null	Perfect	Broaden	Baseline	Intensity	Shift	Impurities
Cubic	51.49	7.55	8.62	71.26	7.84	7.53	59.22
Hexagonal	47.37	7.35	10.92	57.87	7.53	7.47	48.36
Trigonal	46.58	15.72	19.03	41.14	15.97	15.97	39.25
Tetragonal	48.77	11.56	17.15	43.02	11.74	11.62	42.02
Orthorhombic	29.94	10.06	18.45	34.85	10.45	10.02	27.34
Monoclinic	24.76	11.79	20.01	38.21	12.16	11.74	22.94
Triclinic	20.06	3.11	19.87	24.11	3.46	3.15	16.02

**Table 4:** MAPE for 1D-CNNs trained and tested on modified data Incorporating modifications into the training set reduces the MAPE for baseline noise, broadening and multiphase impurities. Null refers to predictions based on the mean lattice parameters of the data set. Perfect refers to training and testing on unmodified data and is intended as a control.

Symmetry	Null	Perfect	Broaden	Baseline	Impurities
Cubic	51.49	7.55	3.4	5.2	9.4
Hexagonal	47.37	7.35	8.0	10.2	13.6
Trigonal	46.58	15.72	15.8	16.9	22.9
Tetragonal	48.77	11.56	12.6	15.6	18.2
Orthorhombic	29.94	10.06	10.3	11.5	17.46
Monoclinic	24.76	11.79	12.4	12.3	16.02
Triclinic	20.06	3.11	3.7	4.6	10.48

**Table 5:** MAPE for 1D-CNNs trained on modified data and tested on unmodified data Prediction is generally worse relative to the perfect condition. However, some classical ML augmentation improvements are apparent for the broadening condition. Null refers to predictions based on the mean lattice parameters of the data set. Perfect refers to training and testing on unmodified data and is intended as a control.

Symmetry	Null	Perfect	Broaden	Baseline	Impurities
Cubic	51.49	7.55	3.1	4.4	9.9
Hexagonal	47.37	7.35	8.5	10.0	13.5
Trigonal	46.58	15.72	16.7	15.5	20.0
Tetragonal	48.77	11.56	13.0	16.5	17.5
Orthorhombic	29.94	10.06	9.5	10.3	15.71
Monoclinic	24.76	11.79	12.6	11.6	13.5
Triclinic	20.06	3.11	3.1	3.8	7.5

**Table 6:** MAPE for 1D-CNNs as a function of the number of visible peaks for models trained on both 0–30° and 0–90° 2θ for the tetragonal crystal system The noise level value indicates the fraction of the largest reflection. For example, 0.05 corresponds to baseline noise which does not exceed 5% of the largest peak. ML full corresponds to the prediction from the 1D-CNN models for 0–90° in 2θ. ML reduced corresponds to the prediction from the 1D-CNN models for 0–30° in 2θ.

Noise level	Percentage of visible peaks	Null	ML prediction full	ML prediction reduced
0.0	100.0	46.58	11.56	9.09
0.001	85.3	46.58	13.56	12.29
0.005	73.4	46.58	14.76	13.38
0.01	67.0	46.58	14.12	14.19
0.05	44.90	46.58	17.21	18.35
0.1	33.30	46.58	19.12	19.15

**Table 7:** The average number of impurity peaks for each intensity range of a given PXRD pattern The intensity level value indicates the fraction of the largest reflection. For example, 0.05 implies that the largest impurity peak does not exceed 5% of the largest peak in the original PXRD pattern.

Intensity level	Average number of peaks
0–0.001	363.3
0.001–0.005	104.0
0.005–0.01	19.1
0.01–0.05	18.9
0.05–0.1	3.7

**Table 8:** Showing how the 1D-CNN models trained on the ICSD/CSD database significantly outperform null predictions PWB reports the percentage of testing examples which have all three length parameters within a given MAPE bound. PWB10, for example, indicates the percentage of testing examples for which all three predicted lattice parameters are within 10% of their true lattice parameters.

	Cubic		Hexagonal		Trigonal		Tetragonal		Orthorhombic		Monoclinic		Triclinic	
PWB	ML	Null	ML	Null	ML	Null	ML	Null	ML	Null	ML	Null	ML	Null
PWB1	19.1	1.3	1.9	0.0	1.4	0.0	0.8	0.0	0.0	0.0	0.0	0.0	1.1	0.1
PWB5	78.3	2.9	34.1	0.0	15.0	0.0	15.5	0.0	9.6	0.0	5.9	0.0	50.1	0.6
PWB10	91.3	15.4	60.7	0.0	32.3	0.0	35.2	0.0	34.5	1.1	23.6	1.8	83.4	4.4
PWB20	96.6	23.8	79.6	0.6	53.9	2.0	56.3	2.4	64.5	10.8	51.0	14.9	93.9	28.5
PWB30	98.1	32.4	86.1	15.5	67.0	8.3	70.9	8.8	80.3	29.7	69.9	36.7	97.7	54.2
PWB40	98.6	44.0	90.7	15.5	75.3	18.1	81.9	21.0	87.8	50.1	83.7	58.0	98.8	72.7
PWB50	99.6	50.8	94.3	29.4	82.4	32.4	88.1	35.8	93.6	64.8	90.6	73.3	99.7	83.0

**Table 9:** Volume of search space ratio for each crystal system

Symmetry	PWB10	PWB5	VR_10_	VR_5_
Cubic	91.3	78.3	369.1	2926.8
Hexagonal	60.7	34.1	139.4	557.7
Trigonal	32.3	15.0	242.5	969.4
Tetragonal	35.2	15.5	181.9	727.5
Orthorhombic	34.5	9.6	3908.7	31 369.3
Monoclinic	23.6	5.9	2139.1	17 017.4
Triclinic	83.4	50.1	1902.1	15 216.7

**Table 10:** Description of case studies for automatic experiments using *Lp-Search*

Example	Structure	Crystal system	Lattice parameters (Å, °)
1	F_0.5_Ga_1.8251_Mg_0.9975_O_3.5_	Cubic	8.292, 8.292, 8.292, 90, 90, 90
2	C_116_H_1_O_4_·4CH_4_O·4H_2_O	Triclinic	11.2927, 13.455, 37.9436, 83.672, 89.873, 80.841
3	C_48_H_62_ErN_7_O_2_Si_2_	Hexagonal	13.1144, 13.1144, 57.64, 90, 90, 120

**Table 11:** Time taken and percentage converged for Example 1 using the ML+*Lp-Search* method Automatic performance is similar to that obtained using default *Lp-Search*. Converged refers to the fraction of time that *Lp-Search* converges to the true lattice parameters in 20 minimizations of 50 000 iterations. VR is the search space volume ratio for each lattice parameter bound.

Lattice parameter range	Converged	〈Time〉 (s)	σ(Time) (s)	VR
10% bound	1.0	0.12	0.02	7.85
20% bound	1.0	0.204	0.025	3.93
50% bound	1.0	0.21	0.0	1.57
(3–2*d*_max_)	1.0	0.28	0.07	1

**Table 12:** Time taken and percentage converged for Example 2 using the ML+*Lp-Search* method ML+*Lp-Search* is considerably faster than default *Lp-Search* performance. Converged refers to the fraction of time that *Lp-Search* converges to the true lattice parameters in 20 minimizations of 50 000 iterations. VR is the search space volume ratio for each lattice parameter bound.

Lattice parameter range	Converged	〈Time〉 (s)	σ(Time) (s)	VR
10% bound	1.0	37.00	27.79	7239
20% bound	1.0	97.97	91.53	920
50% bound	1.0	847.22	860.60	59
(3–2*d*_max_)	1.0	3989.61	3297.83	1

**Table 13:** Time taken and percentage converged for Example 3 using the ML+*Lp-Search* method ML+*Lp-Search* is considerably faster and converges more often than default *Lp-Search*. Converged refers to the fraction of time that *Lp-Search* converges to the true lattice parameters in 20 minimizations of 50 000 iterations. VR is the search space volume ratio for each lattice parameter bound.

Lattice parameter range	Converged	〈Time〉 (s)	σ(Time) (s)	VR
10% bound	1.0	44.8	38.5	404
20% bound	0.85	185.6	142.5	101
50% bound	0.50	354.2	155.1	16
(3–2*d*_max_)	0.0			1

**Table 14:** ML+*Lp-Search* method applied to predict lattice parameters on synchrotron data automatically An asterisk (*) next to a prediction indicates that the predicted lattice parameter is an integral multiple or divisor of the true lattice parameter.

Material	Crystal system	Real *a*	Real *b*	Real *c*	ML *a*	ML *b*	ML *c*	ML/*Lp* *a*	ML/*Lp* *b*	ML/*Lp* *c*
LaB_6_	Cubic	4.1568	4.1568	4.1568	4.0950	4.0950	4.0950	4.1584	4.1584	4.1584
SiO_2_ †	Trigonal	4.9142	4.9142	5.4057	4.6304	4.6304	7.4401	4.9030	4.9030	5.4448
(C_4_H_5_KO_6_)_*n*_ ‡	Orthorhombic	7.6130	7.7872	10.6546	6.8423	9.8300	11.5349	7.6128	7.7871	10.6544
ZnO	Hexagonal	3.2483	3.2483	5.2041	3.1494	3.1494	5.5284	3.2485	3.2485	5.2044
In_2_O_3_	Cubic	10.1146	10.1146	10.1146	10.1178	10.1178	10.1178	10.1152	10.1152	10.1152
Fe_2_O_3_ §	Hexagonal	5.0329	5.0329	13.7420	4.1460	4.1460	9.5030	5.0329	5.0329	6.8710*
CaCO_3_	Hexagonal	4.9865	4.9865	17.0609	4.8977	4.8977	8.9315	4.9876	4.9876	8.5330
NaHCO_3_	Monoclinic	3.4800	9.6844	8.0555	5.4356	6.9519	9.4572	6.7952	12.2167	7.9012
NaCl	Cubic	5.6411	5.6411	5.6411	5.0638	5.0638	5.0638	5.6448	5.6448	5.6448
KCl	Cubic	6.2933	6.2933	6.2933	6.6645	6.6645	6.6645	6.2978	6.2978	6.2978
Na_2_S_2_O_3_ 5H_2_O	Monoclinic	5.9501	7.5349	21.6000	6.4716	10.2068	15.2701	5.9553	15.2145	22.0355
MgCl_2_ 6H_2_O	Monoclinic	6.0748	7.1084	9.8619	4.8602	6.5877	7.5698	6.0751	7.1089	9.8626
(CH_6_N)_2_PbI_3_Cl¶	Orthorhombic	4.6447	15.4300	19.2880	7.3973	14.7956	23.1625	9.2914*	15.4322	19.2914
KHCO_3_	Monoclinic	3.7131	5.6299	15.1794	4.7047	9.6333	16.1360	6.2625	9.8191	24.1920
C††	Cubic	3.5656	3.5656	3.5656	2.7742	2.7742	2.7742	3.5655	3.5655	3.5655

† α-Quartz.

‡ Naturally occurring potassium bitartrate.

§ Contains 7.5 wt% Fe_3_O_4_ impurity.

¶ Contains 5.4 wt% of methylammonium chloride and 0.7 wt% of (CH_6_N)PbI_3_ impurities (Kim *et al.*, 2020 ▸).

†† Diamond.
