# Supplementary material for: Automated prediction of lattice parameters from X-ray powder diffraction patterns
Source: J Appl Crystallogr. 2021 Nov 30;54(Pt 6):1799–810. doi: 10.1107/S1600576721010840 (PMC8662964; doi:10.1107/S1600576721010840)
Supplement: Supplementary file 1 [file j-54-01799-sup1.pdf]

JOURNAL OF
APPLIED
CRYSTALLOGRAPHY

Volume 55 (2022)

Supporting information for article:

Autonomous prediction of lattice parameters from X-ray powder diffraction patterns

Sathya R. Chitturi, Daniel Ratner, Richard C. Walroth, Vivek Thampy, Evan J. Reed, Mike Dunne, Christopher J. Tassone and Kevin H. Stone

Supporting Information: Autonomous Prediction of Lattice Parameters from X-ray Powder Diffraction Patterns

S1. Supplementary Analysis

S1.1. Sorting a, b, c length parameters

In general, we found that the 1D-CNN models converged more quickly when a, b, c were first sorted. For crystal systems such as the orthorhombic system, such a transformation is necessary as the label ordering is not unique. For cases where symmetry conditions required that $a = b$, the a and b lattice parameters were reported as $\frac{a+b}{2}$ or $\frac{c+b}{2}$, depending on whichever of $|b - a|$ and $|c - a|$ was smaller. For instance, if the true lattice parameters were $[21.01, 21.01, 7.18]$, then the ML models are trained with $[7.18, 21.01, 21.01]$ as the label. Now suppose the ML prediction was $[7.41, 22.51, 21.11]$, then in order to enforce $a = b$, the prediction is taken to be $[7.41, 21.81, 21.81]$. Here, another valid approach is to choose an ordering scheme such that the labels always have the repeated parameters first: i.e. $[a, a, c]$. We found that training with this ordering also gave good predictions, however the 1D-CNN models took much longer to train.

S1.2. Challenges with predicting α, β, γ angle parameters

We observed that it is difficult to make good predictions on the angle lattice parameters α, β, γ. We do not understand why this is the case and offer two possible explanations to consider in future work. The first is that there are likely many structures which generate similar diffraction patterns, even after using a reduced cell case. In other words, there are multiple local minima around the global minima of the fitting landscape. If this is the case, potential future work should improve upon the model formulation. One possibility is to embed a PXRD simulator into the neural network and use the loss between predicted patterns and the true pattern to train the model.

This would likely force the model to learn a physical relationship between the lattice parameters. Such an approach follows a paradigm known as Physics-Inspired Neural Networks (Pi-NNs) which use differentiable simulators to encode physical constraints into neural networks.

Another possibility is that the training data overwhelms the model. For the case of angle predictions, for both the triclinic and monoclinic system, the majority of structures have angles very close to 60, 90 and 120. This could bias the model to replicate training data and give incorrect predictions. Future work to investigate this problem could involve simulating additional structures with a wide range of angles and determining whether ML based models can be successful.

Due to these problems, in this analysis we do not try to determine the angles for the monoclinic and triclinic crystals. However, this problem can be alleviated slightly by using *Lp-Search* which can help find the correct angles, as long as the length parameters are initialized well.

S1.3. Null Model Analysis

In this section, we compare the performance of other possible choices for a Null model for lattice parameter prediction. The first Null model we consider is the mean Null model which uses the average lattice parameters of the training dataset as the predicted lattice parameters. A similar model is the median Null model which uses the median values of the training lattice parameters for the prediction. We also investigated two models which use the data in order to make a prediction. The first is the d_{max} model which uses the d value from the first observable PXRD peak as the prediction for all three length lattice parameters. The second data based Null model uses the d value for the c lattice parameter and the mean lattice parameters for a and b. The performance of each Null model is detailed in Table S1 for each crystal system.

Table S1. *Mean Absolute Percentage Error (MAPE) of various Null models for the length lattice parameter prediction task. The performance of the Null model depends on the crystal system under investigation.*

**Crystal System**	**Mean**	**Median**	$\mathbf{d}_{\max}$	**Mean** + $\mathbf{d}_{\max}$
Cubic	51.49	47.55	26.03	43.01
Hexagonal	47.37	43.70	48.50	41.32
Trigonal	46.58	58.84	45.50	44.00
Tetragonal	48.77	57.13	45.76	43.56
Orthorhombic	29.94	35.42	41.00	26.41
Monoclinic	24.76	25.70	46.47	25.53
Triclinic	20.06	20.80	61.63	31.12

Unsurprisingly, the data based models perform well for the cubic crystal system case. However, they are much worse than the data agnostic models for the lower symmetry cases. In short, the different Null models are roughly comparable and the performance of any given model depends on the crystal system.

S1.3.1. Training CNN models on each space group

In this section, we highlight the possible improvements to ML predictive performance when the space group is known and there is sufficient data in at least one space group of a given crystal system. Here, we consider space groups within the monoclinic crystal system since some of these classes still contain a relatively large amount of training data. We train a baseline 1D-CNN on the monoclinic space group with the largest amount of training data (Space group 14) and use a transfer learning approach for the other space groups. Specifically, we initialize neural networks for all other space groups with the trained weights from the most prevalent space group. This procedure is a transfer learning technique known as warm-starting and can be used to increase the speed of neural network training as well as the predictive power on small datasets. Concretely, since the less prevalent space groups only contain a small amount of data, the warm-start procedure allows information sharing from the dominant space group in order to improve predictive power in the small data regime.

Table S2. *Mean Absolute Percentage Error (MAPE) and Percentage Within Bound (PWB) for 1D-CNNs trained on the top 5 monoclinic space groups.*

**Space group Number**	**ML Prediction**	**PWB10**	**PWB5**	**PWB1**	**Dataset Size**
All	11.79	23.6	5.9	0.0	445708
14	7.44	57.5	21.4	0.2	287663
15	7.79	49.8	16.2	0.2	75703
4	4.74	76.2	40.8	1.3	35330
12	10.64	39.5	13.6	0.4	7333
9	9.36	42.1	12.6	0.1	7275

Based on this analysis, we find that the 1D-CNN models for the top 5 space groups (by training dataset volume) have lower MAPE and higher PWB1, PWB2, PWB5 than a single model trained on data from the entire monoclinic crystal system (Table S2). This finding suggests that it is possible to train models for each space group, even in the small data regime, as long as there is enough data for at least one dominant space group. Furthermore, it shows a direct path to improving the performance of ML based models trained on raw intensity profiles. Although, again, it is worth emphasizing that this approach requires more prior knowledge than knowledge of just the crystal system. Nevertheless, we expect this type of analysis to be useful in combination with other CNN based models which seek to predict the space group directly from raw intensity arrays (Park *et al.*, 2017; Vecsei *et al.*, 2019; Oviedo *et al.*, 2019; Suzuki *et al.*, 2020; Tiong *et al.*, 2020).

S2. Supplementary Methods

S2.1. Dataset Description and Simulation

PXRD patterns were simulated from CIF files contained in the ICSD and CSD databases using the CSD Python API (Groom *et al.*, 2016). CIF files were filtered such that only unique, crystalline structures were kept; two structures were designated as different if they had either a different chemical formula, space group or set of lattice parameters. Each pattern was simulated in the range [0, 90] in 2θ with a spacing of 0.01 and an incident wavelength of 1.540 56 Å. These choices correspond to a q range of [0, 6] Å^{-1}. All patterns were normalized such that the intensities were in the [0,1] range. The chosen peak shape was pseudo-Voigt with a Full-Width Half Max of 0.1 2θ and a mixing parameter of 0.5. In total, 961960 patterns were simulated.

S2.2. 1D-CNN Architecture, Training Time and Hyperparameters

In the experiments involving the full 0-90 range, we used the 1D-CNN model described in Table 1. For the 0-30 range, we used the same model and removed the first max pooling operation which reduced the dimensionality of the inputs by a factor of 3. The intention of this procedure was to keep the number of neural network parameters the same to facilitate comparison between the two ranges. In addition, the same hyperparameters were used for all experiments. Specifically, the following values were used: learning rate = 0.001, loss = Huber, batch size = 64, Adam optimizer with $\beta_1 = 0.9$ and $\beta_2 = 0.999$, and Glorot initialization. To select the final model, we performed coarse hyperparameter optimization and considered CNN architectures with variable layer, filter and pool sizes as well as models with skip-connections and batch-normalization. We found that different models worked better on different crystal systems. In order to keep the analysis simple, we chose to use the same model for all crystal systems.

Models which required data augmentation were trained on a CPU with a 2.3 GHz 8-Core Intel Core i9 processor using 16 GB RAM. Model training took approximately 10 hours for the largest training set. Models without data augmentation were trained using 1 GeForce RTX 2080 Ti GPU. Training took approximately 1 hour for the largest training set.

Two custom metrics were used to evaluate our models: Mean Absolute Percentage Error (MAPE) and Percentage Within Bound (PWBX). MAPE, measures the mean absolute percentage difference between $[a, b, c]_{true}$ and $[a, b, c]_{predicted}$. PWBX is the fraction of examples for which each of the true lattice parameters lie within a $X\%$ bound of the corresponding ML predictions. For example, PWB50 is the percentage of testing examples for which each of the true lattice parameters lie within 50% of the corresponding estimated lattice parameter. All models were defined using the Keras Tensorflow API (Chollet *et al.*, 2015).

S2.3. Description of modification Schemes

Some of the analysis in this paper involved the application experimental modifications to either or both of the training and testing sets. In general, data modification (augmentation) is widely used in ML to improve the generalizability of ML models (Perez & Wang, 2017). In contrast to previous work (Oviedo *et al.*, 2019; Park *et al.*, 2017), but consistent with general deep learning guidelines, experimental modification in this analysis is applied during training to each minibatch of data.

- **Random Intensity Modulation**: Each 100 1D-pixel region (90 regions for each pattern) is scaled by a constant factor uniformly drawn from -30% and 30%. Blocks of regions were chosen in order to add more systematic noise as well as to speed up the computational implementation. This modification is intended to coarsely mimic preferred orientation effects.
- **Linear Combination of Phases**: Up to three extra random PXRD patterns are added to each input. The exact number of impurities is drawn from a discrete uniform distribution for each training point. Specifically, each dominant powder

pattern may have 1, 2 or 3 additional weaker intensity patterns. The impurities are scaled by a uniform number $\in [0.05, 0.1]$. This ensures that no impurity peak has an intensity greater than $1/10^{\text{th}}$ of the largest peak in the main structure. This modification is used to simulate the effect of low intensity impurities. In this analysis, the impurity PXRD patterns were selected randomly from any possible crystal system. For example, under this model, it is possible to have a dominant cubic phase, and a number of low symmetry impurities.

- **Gaussian Baseline Noise**: For the general modification experiments, the following noise model was used: $x \sim \mathcal{N}(0,1)\mathcal{U}(0,0.002)$. This model chooses a random noise from a uniform distribution between 0 and 0.002 and modulates this noise by a standard normal distribution. This modification is intended to simulate detector baseline noise. For the investigation of increasing background, gaussian noise is drawn from the distribution $x \sim \mathcal{N}(0, \frac{\text{noise level}}{3})$. This ensures that the noise is below the specified noise level threshold 99.7% of the time.
- **Gaussian Peak Broadening**: A gaussian filter with σ^2 uniformly sampled from [1, 5] is applied to the full PXRD pattern. Here, all data in one minibatch experiences the same broadening factor. This simplification greatly increases the implementation speed. This modification simulates both sample and detector effects.
- **Detector Zero-shift**: A random uniform shift between [-15, 15] 1D-pixels is applied to every peak in a given PXRD pattern; this corresponds to a [-0.01, 0.01] shift in q and a [-0.17, 0.17] shift in 2θ. This modification is also applied the same to all examples within one minibatch. This modification simulates detector offset.

S2.4. Model Training and Testing

For the analysis of the combined ICSD/CSD datasets, datasets of sizes 32705, 19842, 27784, 39183, 163087, 447708, 245651 were simulated for the cubic, hexagonal, trigonal, tetragonal, orthorhombic, monoclinic and triclinic crystal systems respectively according to the procedure in Section S2.1. Models were trained to predict the a, b, c lattice parameters. In order to stabilize the training procedure, the a, b, c were first sorted. The dataset split was such that the testing and validation sets each had 1000 entries. All models were trained for 50 epochs with an early-stopping patience of 20. The MAPE metric was used to select models.

For the experiments involving realistic non-idealities, in order to isolate the effect of one particular experimental modification all other modifications were turned off during

training and testing. Furthermore, for the particular experimental modification, we controlled the four following combinations of training and testing on data with and without the added condition. Note, the experimental modifications applied during training and testing have a random element. For this reason, when testing on any dataset which used experimental modification, the results were averaged over 100 independent predictions on the testing set.

S2.5. Volume of Search Space

Models trained on ICSD/CSD data, with no experimental modifications, were used to quantify the reduction in parameter search space volume (VR) obtained using ML for 1000 testing examples from ICSD/CSD. The VR is calculated as:

$$\mathrm{VR} = \frac{\text{Default Search Space Volume}}{\text{ML Search Space Volume}} \tag{1}$$

The default search space volume is first calculated by determining the range [3, $2d$ $_{\max}$]. Here, d $_{\max}$ is calculated by calculating all d spacings consistent with a given crystal system and lattice parameters. The ML search space volume is calculated by determining $[a_{\max} - a_{\min}]$, $[b_{\max} - b_{\min}]$, $[c_{\max} - c_{\min}]$ which are the edge values of the $X\%$ bound around the predicted a, b, c lattice parameters. Thus, the VR is written as:

$$\mathrm{VR} = \begin{cases} \frac{|2d_{max}-3|}{(a_{\max}-a_{\min})}, & ;\ \mathrm{a} = \mathrm{b} = \mathrm{c} \\ \frac{|2d_{max}-3|^2}{(a_{\max}-a_{\min})(c_{\max}-c_{\min})} & ;\ \mathrm{a} = \mathrm{b} \neq c \\ \frac{|2d_{max}-3|^3}{(a_{\max}-a_{\min})(b_{\max}-b_{\min})(c_{\max}-c_{\min})} & ;\ \mathrm{a} \neq b \neq c \end{cases}$$

Note, the minimum and maximum values depend on the bound chosen for the ML prediction. For clarity, a subscript is added to VR to indicate the bound. In this

analysis, VR_5 and VR_{10} are reported.

S2.6. Lp-Search

The 1D-CNN models used for the VR calculations were used to make predictions on 3 structures within the testing data corresponding to a high symmetry system, a low symmetry system and a dominant zone system (Table 10). These predictions were used to calculate the corresponding $[a_{\max} - a_{\min}]$, $[b_{\max} - b_{\min}]$, $[c_{\max} - c_{\min}]$ for a 10% bound and a 50% bound. These ranges, the corresponding PXRD patterns, and the lowest symmetry space group compatible with correct crystal class were input into *Lp-Search*. A heuristic unit-cell volume range $[0.5abc,\ 1.5abc]$ was also specified for each PXRD pattern. The minimizations were terminated if the $Rwp < 2$ and the total unit-cell volume was correct to within 1% or if the algorithm reached 50000 iterations. Experiments were repeated 20 times and average time and fraction of times converged were reported. The ML+*Lp-Search* analysis was compared against a baseline where $3 - 2d_{max}$ was used for the initial *Lp-Search* range.

S2.7. ML+Lp-Search for Experimental Data

For the experimental data validation on synchrotron data from SSRL, a sequential procedure of ML and *Lp-Search* was used. In order to make ML predictions, the SSRL dataset was baseline corrected and converted to the q range 0 to 6 to match the ICSD/CSD data. For non-zero baselines, we used the dual-tree complex waveform algorithm for autobaseline removal (René de Cotret & Siwick, 2017). In addition, the data was linearly interpolated, where required, to match the input length in the ICSD/CSD data. This step was necessary since the 1D-CNN models in our analysis only accept a fixed size input. For each experimental pattern, the *Lp-Search* minimization procedure was run with the following percentage bounds: 10, 30 and 50%

bound. For each bound, the algorithm was run for 5000 iterations and the solution with the lowest Rwp was chosen. Lattice parameters from ML+*Lp-Search* were compared directly against the ground truth unit-cell solution.

S3. Supplementary Data

In Figures S1-S3 we plot the PXRD patterns for the Examples 1-3 (Table 10) on a square-root intensity scale.

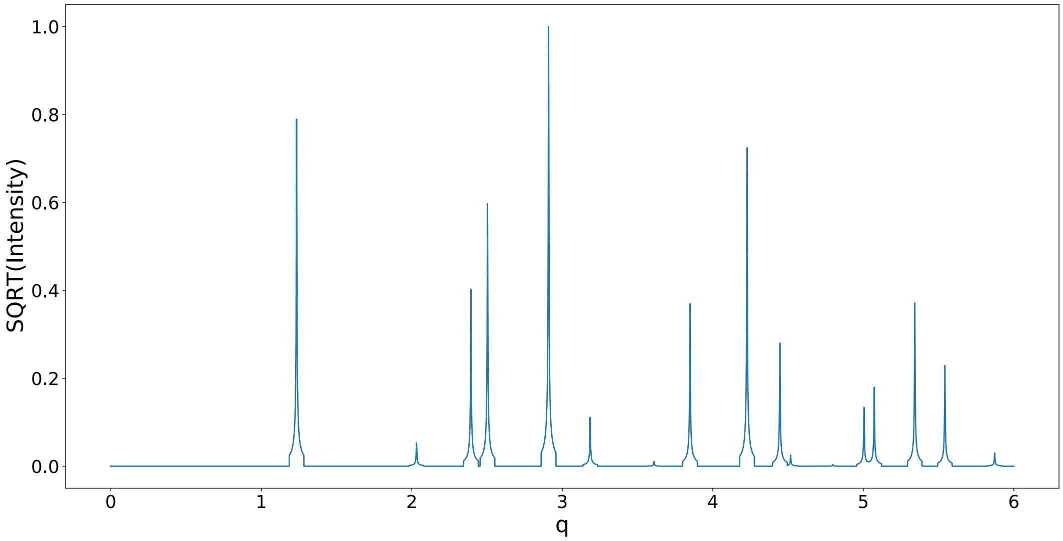

Fig. S1. PXRD pattern for Example 1. This corresponds to a cubic structure with lattice parameter [8.292]. The example was intended to be a simple high-symmetry structure.

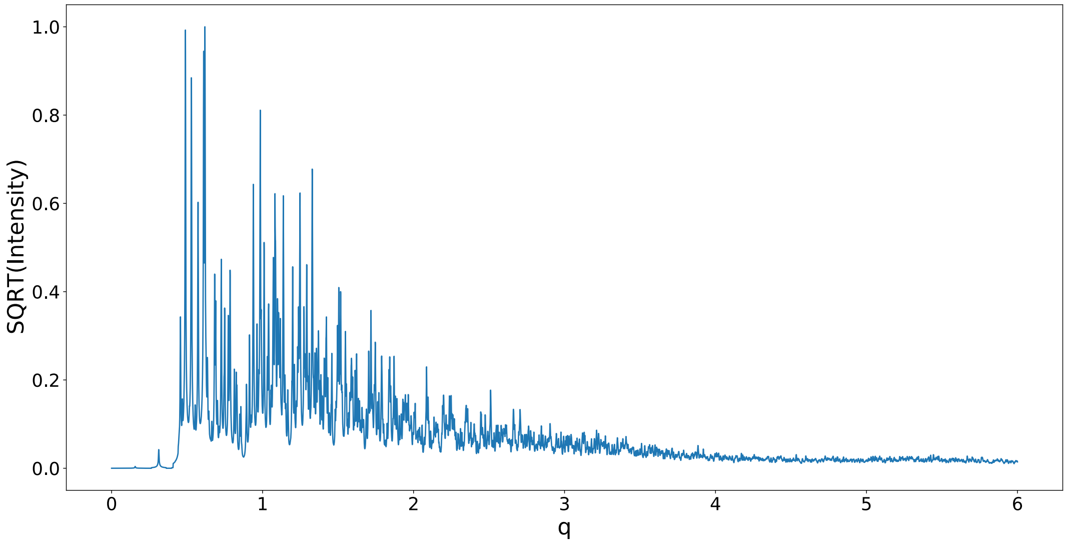

Fig. S2. PXRD pattern for Example 2. This corresponds to a triclinic structure with lattice parameters $[11.2927, 13.455, 37.9436, 83.672, 89.873, 80.841]$. The example was intended to be a relatively challenging low-symmetry structure.

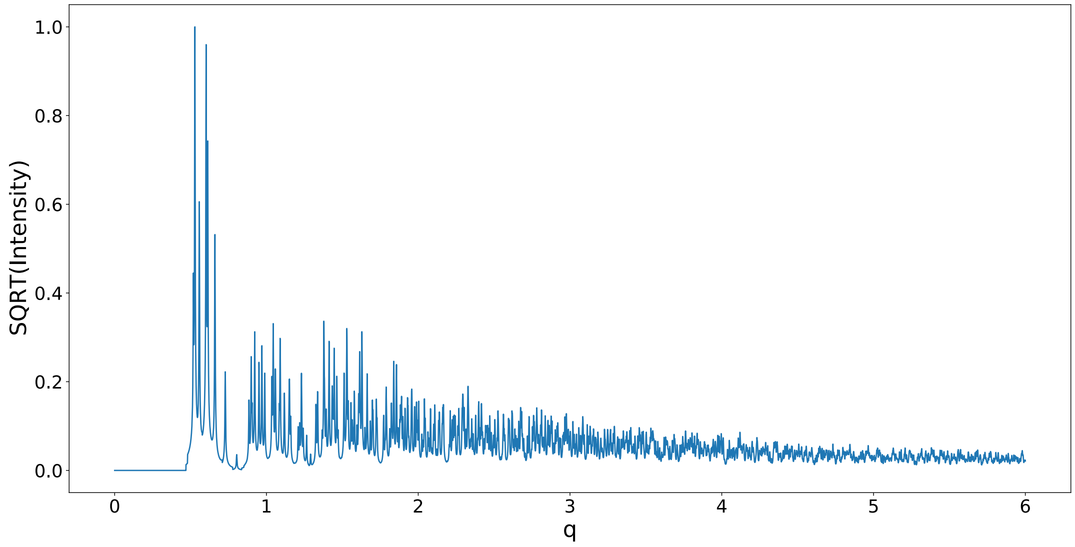

Fig. S3. PXRD pattern for Example 3. This corresponds to a hexagonal structure with lattice parameters $[13.1144, 13.1144, 57.64]$. This example exhibits the dominant zone problem and is challenging for conventional indexing methods.
